# A Combination of Novel Microecological Agents and Molasses Role in Digestibility and Fermentation of Rice Straw by Facilitating the Ruminal Microbial Colonization

**DOI:** 10.3389/fmicb.2022.948049

**Published:** 2022-07-14

**Authors:** Yulin Ma, Xu Chen, Muhammad Zahoor Khan, Jianxin Xiao, Zhijun Cao

**Affiliations:** ^1^State Key Laboratory of Animal Nutrition, College of Animal Science and Technology, China Agricultural University, Beijing, China; ^2^Faculty of Veterinary and Animal Sciences, Department of Animal Sciences, University of Agriculture, Dera Ismail Khan, Pakistan

**Keywords:** microecological agents, molasses, *in vitro* degradability, rumen microbial colonization, rice straw

## Abstract

In this study, we evaluated the effect of microecological agents (MA) combined with molasses (M) on the biodegradation of rice straw in the rumen. Rice straw was pretreated in laboratory polyethylene 25 × 35 cm sterile bags with no additive control (Con), MA, and MA + M for 7, 15, 30, and 45 days, and then the efficacy of MA + M pretreatment was evaluated both *in vitro* and *in vivo*. The scanning electron microscopy, X-ray diffraction analysis, and Fourier-transform infrared spectroscopy results showed that the MA or MA + M pretreatment altered the physical and chemical structure of rice straw. Meanwhile, the ruminal microbial attachment on the surface of rice straw was significantly increased after MA+M pretreatment. Furthermore, MA + M not only promoted rice straw fermentation *in vitro* but also improved digestibility by specifically inducing rumen colonization of *Prevotellaceae_UCG-001, Butyrivibrio*, and *Succinimonas*. Altogether, we concluded that microecological agents and molasses could be the best choices as a biological pretreatment for rice straw to enhance its nutritive value as a ruminant's feed.

## Introduction

Lignocellulosic resources are potential ruminant feed materials with easy availability and low cost. Among lignocellulosic biomass, their compositional contents are highly variable for hemicellulose, cellulose, and lignin, hence their degradability is different. Rice straw is a common lignocellulosic material that is distributed abundantly throughout the globe. According to statistics, the annual output is ~1.14 billion tons (Satlewal et al., 2018). Rice straw is composed of 35.5% cellulose, 25.6% hemicellulose, and 16% lignin (Patel et al., 2020). However, most of the straw is disposed of by incineration, which causes resource wastage and environmental pollution (Zhao et al., 2019). In fact, this is impractical for the direct utilization of rice straw as feed for ruminants due to the highly polymeric and complex lignocellulose structure (Zhang Z. et al., 2016). Hence, pretreatments are essential to overcome the recalcitrance of rice straw to utilize them for better management and generation of bioproducts such as biogas (Kumar et al., 2019).

Previous studies have explored various pretreatment methods, including steam explosion (Shi et al., 2019), alkali (He et al., 2019), and acid (He et al., 2020). Among them, a pretreatment method of microecological agents (MA) composed of *lactobacillus* and cellulolytic enzyme pretreatment ways are currently the most popular technologies in production applications and have shown environmentally friendly performance (Zhang et al., 2021). The low water-soluble carbohydrate (WSC) content in rice straw may limit the MA activity during the fermentation process. This results in the insufficient substrate for MA fermentation to rapidly lower pH and nutrient loss. It is challenging to obtain high-quality silage from rice straw without exogenous WSC (Oladosu et al., 2016). There are frequent and effective ways to improve the WSC content in rice straw by adding cheap sources of molasses (Yuan et al., 2016b). Molasses, a by-product of the sugar industry, is a highly soluble carbohydrate and can be used as a fermentation substrate.

It has been well-documented that more than 90% of lignocellulose is produced by short-chain fatty acids and microbial proteins under the action of rumen microorganisms (Deng et al., 2017). However, the composition of the rumen bacterial community in the anaerobic digestion of lignocellulose has not been deeply studied. Anaerobic digestion is an effective management strategy for microbial pretreatment of waste biomass resources. Up to 95% of organic matter can be produced through anaerobic digestion. It involves the hydrolysis of complex organics into simpler small molecules. Volatile fatty acids (VFAs) produce CH_4_ and CO_2_ gases under anaerobic conditions. The end product of anaerobic digestion is biogas, an energy-rich biofuel (Patel et al., 2021). Previous research trials have reported that *Bacteroidetes, Firmicutes, Fibrobacteres*, and *Proteobacteria* were the main phylum in rumen bacteria, which contributes to the degradation of fiber in the rumen (Ozbayram et al., 2018; Xing et al., 2020). Comtet-Marre et al. (2017) indicated that *Ruminococcus, Fibrobacter*, and *Prevotella* played a pivotal role in polysaccharide degradation (Comtet-Marre et al., 2017). However, the transformation of rumen bacterial community structure and preference of rumen bacterial colonization in MA inoculant pretreatment of lignocellulosic biomass are still unclear.

Thus, this study was designed to investigate the effects of MA ensiling pretreatment on fermentation dynamics, chemical composition dynamics, physical structure, lignocellulosic *in vitro* degradation, volatile fatty acids (TVFA), 72-h cumulative gas production (GP_72_), and the temporal changes in the bacterial communities that colonize the rumen of rice straw.

## Materials and Methods

### Ethical Statement

This study was reviewed and approved by the Animal Protection Professional Committee of the College of Animal Science and Technology, China Agricultural University (Protocol number: 2013-5-LZ).

### Microecological Agents

The microecological agents consist of *lactobacillus* and enzymes. The lactobacillus including *Lactobacillus plantarum* (1.4 × 10^9^ cfu/g) and *L. buchneri* (6 × 10^8^ cfu/g) and the enzymes including *Cellulose* (336 U/g), *Xylanase* (2,080 U/g), and β*-glucanase* (1,920 U/g) was provided by the Feed Research Institute, Chinese Academy of Agricultural Sciences.

### Substrate and Treatment

The rice straw was acquired after the rice harvest on suburban farms (32.13°N, 114.07°E, Gushi County, Xinyang City, Henan province, China) on December 10, 2019. The 500 g of rice straw was weighted and chopped into 3–5 cm lengths and stored in laboratory polyethylene 25 × 35 cm sterile bags provided by Beijing Shengya Yuda Biological Technology Co., Ltd. (Beijing, China); a total of 180 bags of rice straw were prepared. The bags were divided into 3 batches: (1) not treated with any additive (CON), (2) treated with an MA-containing additive (MA), and (3) similar to 2, but with molasses (MA + M; Hebei Shuntong Encyclopedia Trading Co., Ltd., China). The pretreatments were sprayed on their respective rice straw batches according to the 50 g/t rate. The moisture content of the rice straw was adjusted to 70% by adding distilled water and then stored in laboratory polyethylene sterile bags and sealed by a food vacuum sealing machine (Konka KZ-ZK007; Dongguan Yijian Packaging Machinery Co. Ltd., Dongguan, China) and stored at ambient temperature (25 ± 3°C) for 7, 15, 30, and 45 days. Each approach contained 15 bags at each storage stage. Later, the fermentation quality and chemical composition of samples were analyzed on days 7, 15, 30, and 45. In addition, the samples stored for 45 days were used for structural changes and grounded in a hammer mill to bypass through a 1-mm sieve. Then, the *in vitro* digestibility and rumen microbial colonization *in vivo* of the pretreated rice straw was evaluated by passing them through a 2.5-mm sieve, and each was tested in triplicate.

### Chemical Composition and *in vitro* Digestibility Analyses

The dry matter (DM), crude ash (Ash), neutral detergent fiber (NDF), acid detergent fiber (ADF), and crude protein (CP) contents of the rice straw samples were measured using a previously adopted method (Van Soest et al., 1991). The ANKOM 2000i automatic fiber analyzer (Beijing Anke Borui Technology Co. Ltd., Beijing, China) was used to measure the NDF and ADF contents.

An *in vitro* digestibility study was carried out using fluid from rumen samples from three healthy Holstein cows. The samples were collected 2 h after the morning feeding. The rumen fluid was immediately mixed and was kept in a vacuum flask at a 39°C pre-temperature before transportation to the laboratory. Cows were fed a TMR containing 5.6% oat hay, 11.5% alfalfa hay, 8.3% alfalfa silage, and 24.5% corn silage and 50.1% concentrate (13.7% of stem-flaked corn, 5.0% of corn, 8.4% of soybean meal, 5.2% of soybean hull, 4.4% of corn DDGS, 2.9% of molasses, 3.3% of cottonseed meal, 3.3% of sprayed corn skin, 0.5% of Berg + Schmidt, 0.3% of XP XPC, 2.4% of premix, 0.4% of NaHPO_3_, and 0.3% OPTIGEN of DM) and was offered three times per day (07:00, 14:00, and 18.30). Free access to the cows was assured throughout the day.

An automatic trace gas recording system (AGRS) was used to measure the *in vitro* gas production, as described in our previous study (Yuan et al., 2016a). Briefly, 500 mg of rice straw (6 replicates) were placed in 120 ml glass bottles. To each bottle, 50 ml of buffer solution was then added. Under anaerobic conditions, the bottle was filled with nitrogen for 5 s, sealed, connected to the AGRS, and incubated at 39°C for 72 h (Zheng et al., 2021). For each run, there were six bottles for blank correction. The bottles were removed from the AGRS system after 72 h of incubation, and the pH value was immediately recorded. An interphosphate solution of 0.3 ml containing 2.5 g/l was intermixed with 1 ml of culture medium at 4°C for 30 min and centrifuged at 10,000 × g at 4°C for 10 min. For the measurement of acetate acid (AA), propionic acid (PA), butyrate acid (BA), and total volatile fatty acids (VFA), the supernatant was stored at −20°C. The supernatant was removed from the bottle, the residual sample was dried at 65°C, and then DM, NDF, and ADF were measured. *In vitro* disappearance of DM (IVDMD), NDF (IVNDFD), and ADF (IVADFD) was calculated as a difference between the initial culture of DM, NDF, ADF residual DM, NDF, and ADF and was corrected by blanks.

### Structural Analyses

The morphological and structural images of the rice straw before and after treatment with MA or MA + M were acquired using scanning electron microscopy (SEM, ASU 3500, Japan) at a magnification of 1,500. The samples were sputter-coated with platinum to facilitate electrical conductivity. The cellulose crystallinity index (CrI) was determined using the X-ray diffraction (XRD) method (Wu et al., 2021). The XRD was performed using a Siemens D-5000 diffractometer (Bruker, Ettlingen, Germany), and Cu-K radiation was created at 20 mA and 40 kV. Samples were scanned from 3° to 40° with a step size of 0.02 and 3 s per step. The CrI was measured using the following formula:


$$\begin{array}{l} \text{CrI}=\left({\text{I}}_{002}-{\text{I}}_{\text{am}}\right)/{\text{I}}_{002} \end{array}$$

I_002_ represents the scattered intensity at the main peak for cellulose type I. I_am_ represents the scattered intensity due to the amorphous portion, measured as the least intensity between the secondary and main peaks (Segal et al., 1959).

### *In situ* Rumen Incubation

The *in situ* rumen incubation was determined as per the method described by Gharechahi et al. (2020). In brief, pretreated rice straw samples were air-dried and ground using a 2-mm sieve Wiley mill (KRT-34; KunJie, Beijing, China). In addition, 5 g of the milled samples were put into nylon bags (8 x 16 cm; pore size = 50 mm; 6 replicates). The bags were incubated for 0.5, 4, 12, and 24 h in three fistulated cows (Two parallel bags of each pretreated sample were fitted in the cow at a given time point). The bags were removed, washed with sterile saline to remove loosely attached microbiota, and immediately frozen in dry ice and shifted to the laboratory for storage at −80°C until DNA extraction.

### DNA Extraction and Quantitative Real-Time PCR Analysis

Total microbial genomic DNA was extracted from 200 mg of the rumen-incubated rice straw samples using the EZNA Stool DNA kit (Omega Biotek, Norcross, GA, US). The V3–V4 variable region of the 16S rDNA was targeted using primers Eub338F (ACTCCTACGGGAGGCAGCAG) and Eub806R (GGACTACHVGGGTWTCTAAT). Quantitative real-time PCR (qPCR) was carried out following the procedures adopted in a previous study (Jiao et al., 2014). A standard curve was generated from the plasmid DNA of the 16S/18S rRNA gene insert, and the standard curve met the following requirements: *R*^2^ > 0.99. The qPCR assay was performed to generate fragments of 460 base pairs appropriate for paired-end sequencing on the Illumina Miseq system (Shanghai Majorbio Bio-pharm Technology Co., Ltd). The reactions were carried out in a 20 μl mixture containing 7.4% of ddH_2_O, 0.8 μl of each primer (5 μM), 10 μl of 2X Taq Plus Master Mix, and 1 μl of each reaction was used as a template for PCR. Each sample was performed in triplicate for PCR reactions.

### Data Analysis

The cumulative gas production (GP_72_) (ml/g) data were recorded using the AGRS system and fitted to the Groot model as per equation (1) (Jcj et al., 1996).


(1) $$\begin{array}{r} \text{G}{\text{P}}_{\text{t}}=\text{A}/\left[1+{\left(\text{C}/\text{t}\right)}^{\text{B}}\right] \end{array}$$

“A” is the asymptotic gas production (ml/g); “B” is a sharpness parameter determining the curve's shape; “C” is the time (h) at which half of A is reached; and “t” is the *in vitro* incubation time (h).

The effects of the ensiling day, pretreatment, and their interaction were analyzed using a two-way analysis of variance. One-way ANOVA analysis was performed to measure the MA or MA + M pretreatment effect on the structure and *in vitro* digestibility of rice straw. The level of significance was declared at *P* < 0.05. All statistical procedures were performed using SPSS 24 (SPSS Inc., Chicago, IL, USA). The DNA sequencing data were analyzed on a free online platform of Majorbio tools: https://cloud.majorbio.com/page/project/p.html.

## Results

### Physical Structure and Physicochemical Properties

**Figure 1:**
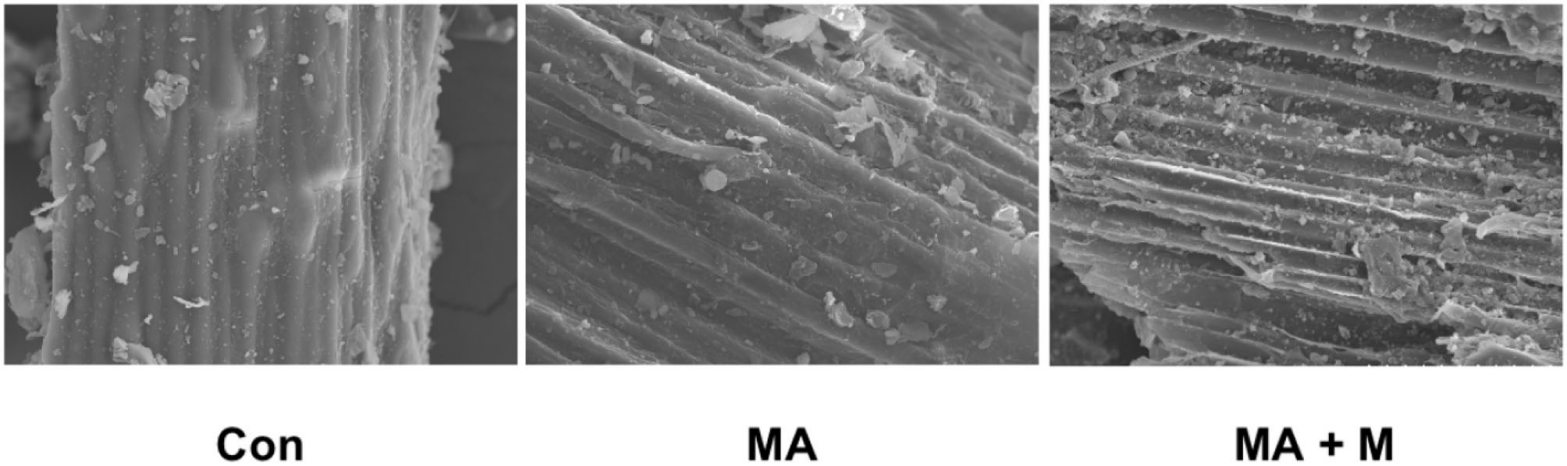
The effect of MA pretreatment on the surface structure of rice straw was analyzed using scanning electron microscopy (SEM). Con, no additive, control; MA, added microecological agents; MA + M, a combination of microecological agents and molasses.

The SEM images of raw material and MA or MA + M pretreated rice straw were used to describe the changes in morphology. After pretreatment with MA or MA + M, the rice straw samples reflected somewhat melted and patchy surfaces (Figure 1), whereas Con exhibited smooth surfaces.

**Figure 2:**
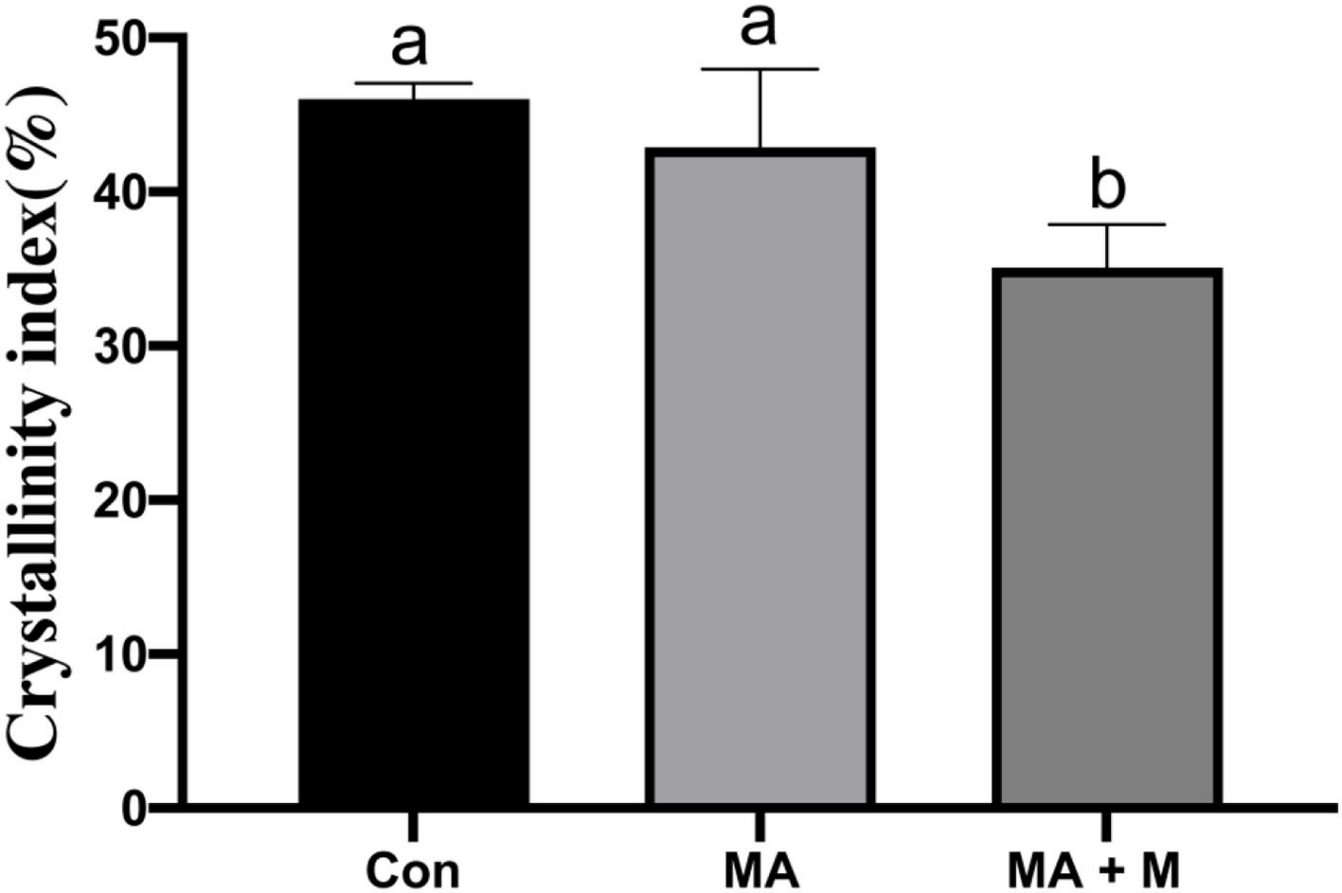
The effect of MA pretreatment on the cellulose crystalline index (CrI, %) of rice straw was analyzed using X-ray diffraction (XRD). Con, no additive, control; MA, added microecological agents; MA + M, a combination of microecological agents and molasses.

To observe the changes in cellulose structure, XRD diffraction data were acquired (Figure 2). The CrI of MA + M was decreased (*P* < 0.05) compared to LAB and Con (Figure 2), and no difference was found between LAB and Con (*P* > 0.05).

**Figure 3:**
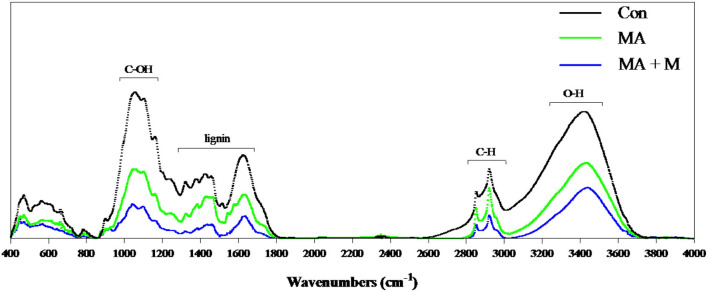
FTIR spectroscopy of rice straw after MA pretreatment. Con, no additive, control; MA, added microecological agents; MA + M, a combination of microecological agents and molasses.

To evaluate the structural and chemical alterations in lignin after pretreatment, the FTIR was performed to detect the lignin extracted from untreated and pretreated rice straw residues (Figure 3). The intensity of the peak at 3,350, 2,900, 1,200–1,000, 1,425, and 1,640 cm^−1^ was reduced after MA or MA + M pretreatment, and the MA + M group was the lowest in each peak.

The NDF (*P* < 0.001) and ADF (*P* < 0.001) contents (Table 1) of rice straw were decreased in MA and MA + M treatments compared to Con, and the MA + M group had the lowest NDF and ADF contents of rice straw (*P* < 0.001). While the content of DM was in MA and MA + M higher than Con (*P* < 0.05), no difference was found between MA and MA + M (*P* > 0.05). The content of CP was higher in MA + M compared to other groups (*P* < 0.001), and the MA content of CP was higher than Con (*P* < 0.05). The EE and Ash contents were decreased in the MA and MA + M groups and the MA + M group had the lowest EE and Ash contents of rice straw (*P* < 0.05). In addition, the CP content of rice straw increased with the extension of anaerobic fermentation days and reached the highest level at 45 days (*P* < 0.001). Meanwhile, after 45 days of ensiling, the NDF (*P* < 0.001) and ADF (*P* < 0.001) contents of rice straw were the lowest. When rice straw was anaerobically fermented for 45 days, the EE content increased compared with the seventh day, while the Ash content decreased (*P* < 0.001).

**Table 1:** Effect of MA pretreatment and anaerobic storage days on the chemical composition of rice straw (%, DM).

**Item**	**Day**				**SEM**	**Treatment**			**SEM**	***P-*** **value**		
	**7**	**15**	**30**	**45**		**Con**	**MA**	**MA + M**		**D**	**T**	**D × T**
CP, %DM	3.06^c^	2.94^d^	3.25^b^	3.32^a^	0.02	2.98^c^	3.14^b^	3.31^a^	0.02	<0.001	<0.001	<0.001
DM, %	96.26^b^	96.84^a^	96.22^b^	96.27^b^	0.03	96.19^b^	96.56^a^	96.46^a^	0.03	<0.001	<0.001	<0.001
NDF, %DM	65.57^a^	60.84^b^	60.11^c^	57.63^d^	0.39	68.82^a^	62.79^b^	56.51^c^	0.27	<0.001	<0.001	<0.001
ADF, %DM	39.32^a^	37.79^b^	35.75^c^	35.46^d^	0.27	40.07^a^	36.44^b^	34.72^c^	0.15	<0.001	<0.001	<0.001
EE, %DM	1.80^b^	1.38^c^	1.92^a^	1.94^a^	0.03	1.44^a^	1.84^b^	2.00^c^	0.02	<0.001	<0.001	<0.001
Ash, %DM	15.87^a^	15.38^c^	15.54^b^	15.69^b^	0.03	15.82^a^	15.64^b^	15.34^c^	0.02	<0.001	<0.001	<0.001

*Different superscript letters a, b, c, and d indicate significantly different values (P < 0.05) across rows, and the same letters indicate insignificant differences (P > 0.05). SEM, standard error of the mean; Con, no additive, control; MA, added microecological agents; MA + M, a combination of microecological agents and molasses; DM, dry matter (the dry matter content is calculated based on air-drying the sample); CP, crude protein; NDF, neutral detergent fiber; ADF, acid detergent fiber; EE, ether extract*.

**Figure 4:**
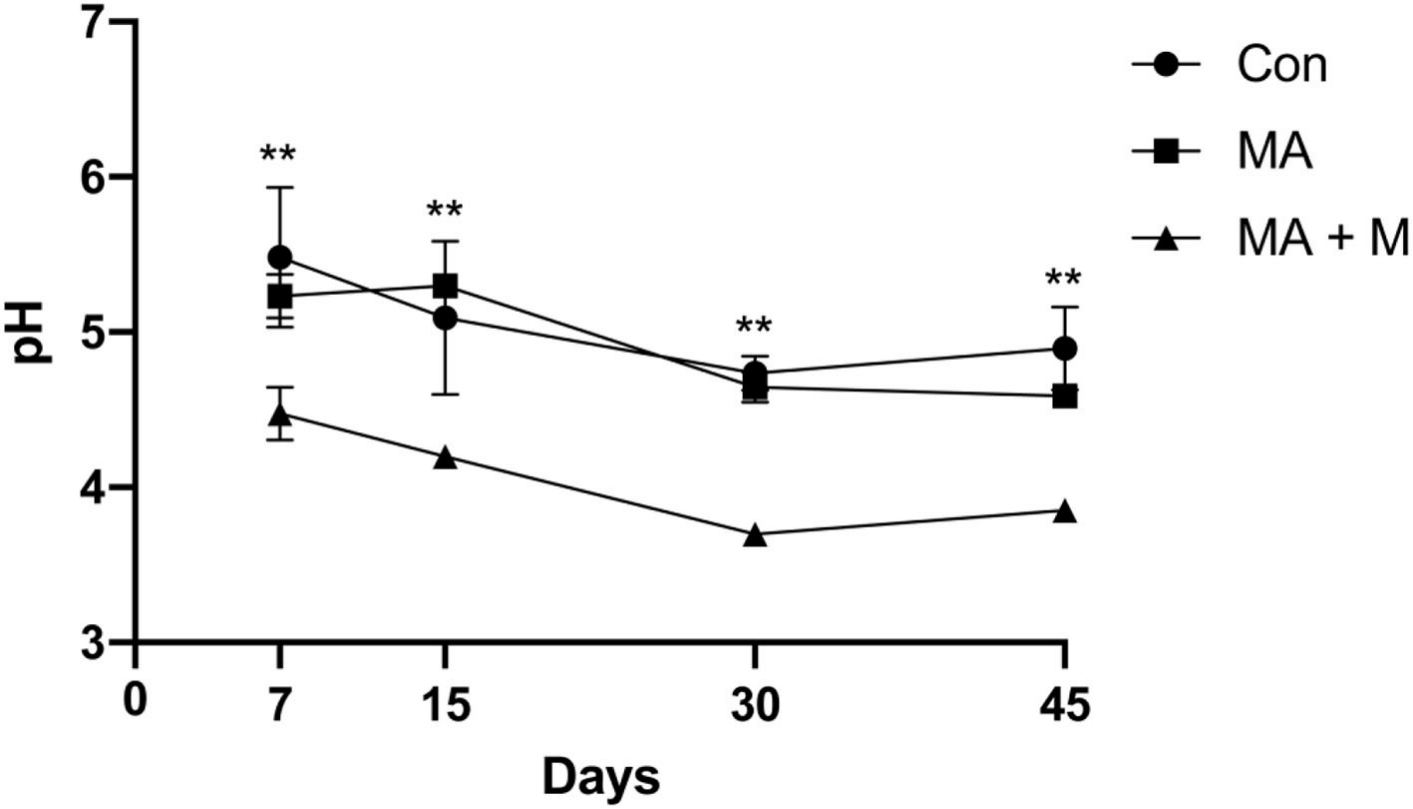
The effect of MA pretreatment on the pH value of rice straw. Con, no additive, control; MA, added microecological agents; MA + M, a combination of microecological agents and molasses. ** indicate the significant correlations at *P* < 0.01.

In this study, with the extension of the fermentation days, the pH value of each group showed a different decrease (Figure 4), and the MA + M (3.7) (*P* < 0.001) had the lowest pH value after ensiling for 30 days.

### *In vitro* Ruminal Degradation and Total Gas Production

The MA + M and MA treatments increased DM (*P* < 0.001), NDF (*P* < 0.001), and ADF (*P* < 0.001) *in vitro* degradation (Table 2) of rice straw compared to Con. The MA + M group was the highest. The MA + M increased gas production (*P* = 0.050) over the 72-h incubation period and asymptotic gas production (*P* = 0.049) compared to MA and Con, while no significant difference was noticed between Con and MA treatments (*P* > 0.05) for gas production and asymptotic gas production. However, the C of the MA + M group was lower than the M group (*P* = 0.012). Notably, the AGPR of the MA + M group was significantly higher than Con and MA groups (*P* = 0.010), while no significant difference was found between the Con and MA treatments (*P* > 0.05).

**Table 2:** Effects of MA pretreatment on the biodegradation and parameters of gas production after 72 h of *in vitro* ruminal incubation of rice straw.

**Item**	**Con**	**MA**	**MA + M**	**SEM**	***P-*value**
**Biodegradation (%)**					
DM degradation	50.40^c^	56.78^b^	60.86^a^	0.56	<0.001
NDF degradation	45.00^c^	48.75^b^	53.20^a^	0.67	<0.001
ADF degradation	40.72^c^	45.15^b^	50.55^a^	0.71	<0.001
**Gas production**					
Total gas (mL/g of DM)					
GP_72_ (mL/g)	145.07^b^	146.29^b^	164.76^a^	4.95	0.050
A (mL/g)	154.65^b^	157.75^b^	176.53^a^	5.7	0.049
B (h)	1.36	1.34	1.31	0.07	0.912
C (h)	8.85^ab^	9.85^a^	7.68^b^	0.34	0.012
AGPR (mL/h)	5.92^b^	5.37^b^	7.52^a^	0.33	0.010

*Different superscript letters a, b, and c indicate significantly different values (P < 0.05) across rows, and the same letters indicate insignificant differences (P > 0.05). SEM: standard error of the mean. Con, no additive, control; MA, added microecological agents; MA + M, a combination of microecological agents and molasses; A, the asymptotic gas production (mL/g DM); B, a sharpness parameter determining the curve's shape; C, the time (h) at which half of A is reached; AGPR, average gas production rate (ml/h)*.

In our current experimental trials, we documented that MA and MA + M significantly (*P* < 0.001) increased the AA and VFA concentrations of rice straw (Table 3). In addition, no significant difference (*P* > 0.05) was found in the VFA and AA concentrations between the MA and MA + M treatments.

**Table 3:** Effect of MA pretreatments on *in vitro* rumen fermentation parameters of rice straw.

**Item**	**Con**	**MA**	**MA + M**	**SEM**	***P*-value**
Acetic acid (mM/L)	40.82^b^	45.81^a^	49.96^a^	0.81	<0.001
Propionic acid (mM/L)	13.39	15.06	14.64	1.43	0.069
Butyric acid (mM/L)	5.34	5.53	5.57	0.88	0.919
Total volatile fatty acid (mM/L)	62.08^b^	69.31^a^	73.08^a^	2.86	<0.001

*Different superscript letters a and b indicate significantly different values (P < 0.05) across rows, and the same or no letters indicate insignificant differences (P > 0.05). SEM: standard error of the mean. Con, no additive, control; MA, added microecological agents; MA + M, a combination of microecological agents and molasses*.

### Microbial Colonization in Pretreated Rice Straw

**Figure 5:**
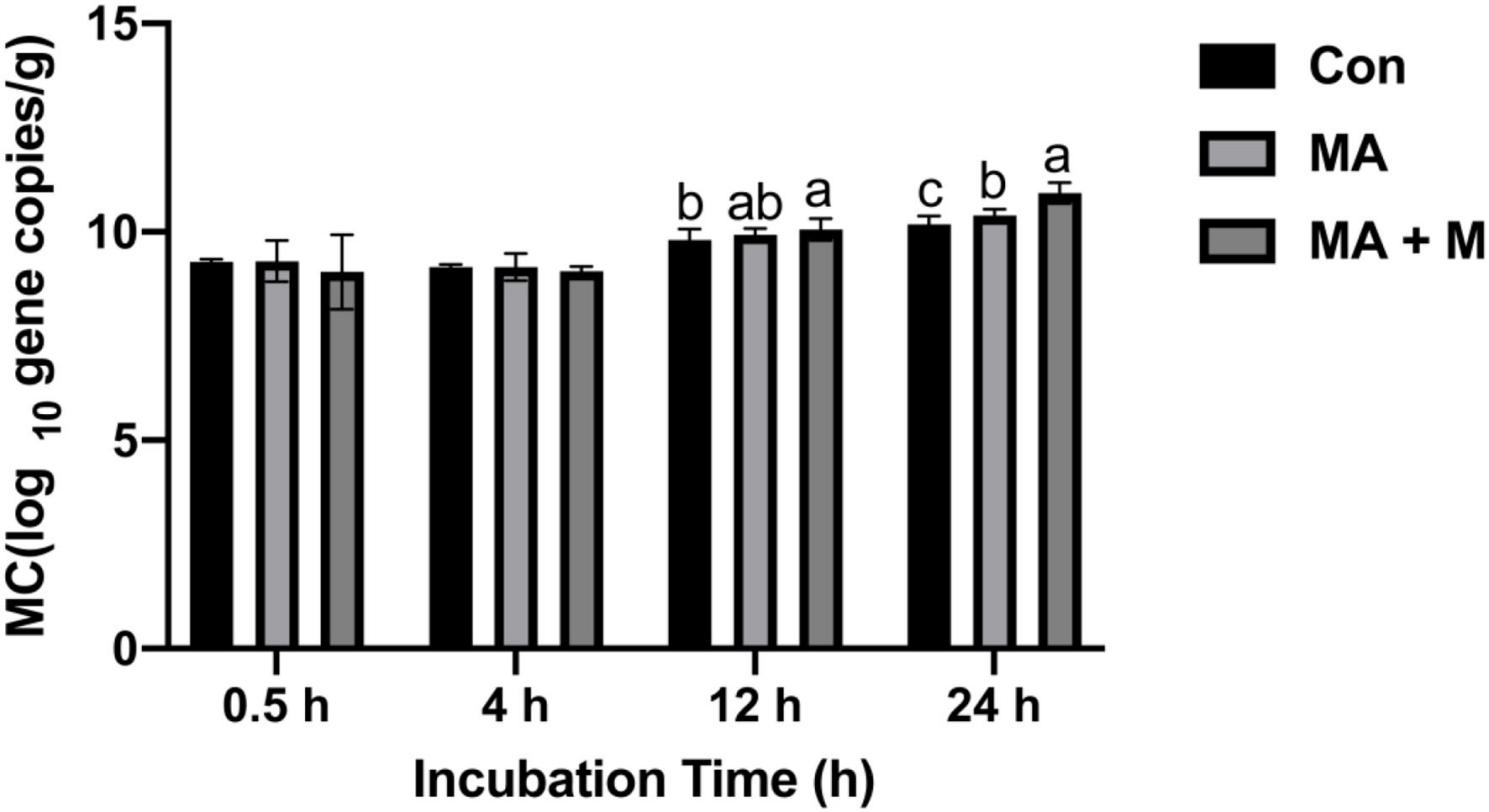
The effect of MA pretreatment on the microbial colonization (MC) of rice straw. Con, no additive, control; MA, added microecological agents; MA + M, a combination of microecological agents and molasses. Different superscript letters a, b, and c indicate significantly different values (*P* < 0.05) in different groups and the same or no letters indicate insignificant differences (*P* > 0.05).

The microbial colonization in the rice straw samples was estimated by measuring the total copy number of bacterial 16S rRNA genes (Figure 5). With increasing incubation time in the rumen, the microbial colonization (MC) significantly increased (*P* < 0.05) in the rice straw. Higher MC (*P* < 0.05) was also observed in the MA + M and MA treatments than in the Con at 12 h of rumen incubation. In addition, the MC of the MA + M group was the highest after 24 days of rumen incubation (*P* < 0.05).

### Diversity of the Bacterial Microbiota Attached to Rice Straw Samples After 0.5 h of Rumen Incubation

**Figure 6:**
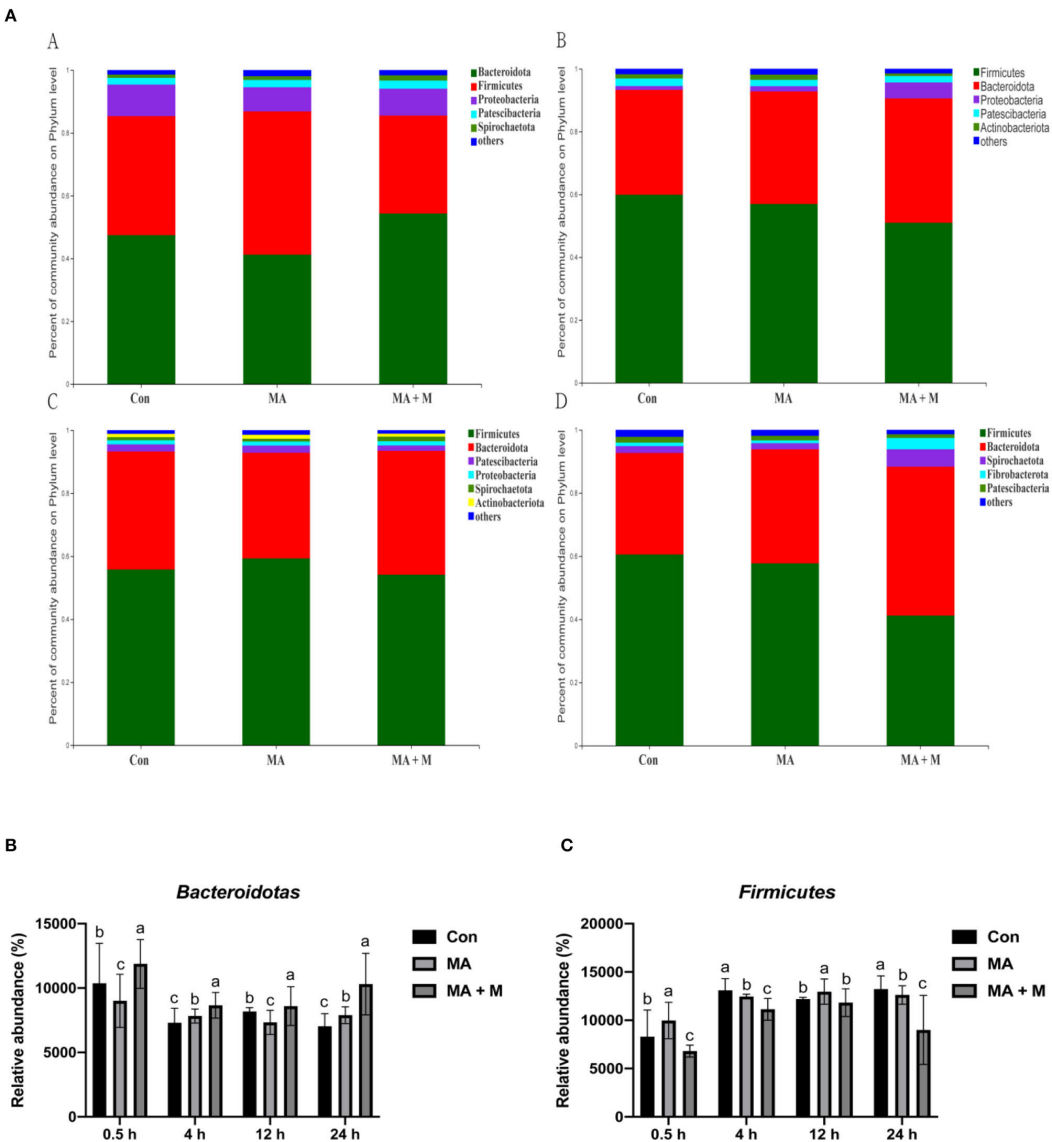
The relative abundance of microbial species under different pretreatment groups. **(A)** Histogram of the relative abundances of species of the rumen microbiome in all pretreatments at the phylum level (abundances <0.01 were grouped as “others”). **(B)** The relative abundance of *Bacteroidotas* in all pretreatments. **(C)** The relative abundance of *Firmicutes* in all pretreatments. Con, no additive, control; MA, added microecological agents; MA + M, a combination of microecological agents and molasses. Different superscript letters a, b, and c indicate significantly different values (*P* < 0.05) in different groups and the same or no letters indicate insignificant differences (*P* > 0.05).

**Figure 7:**
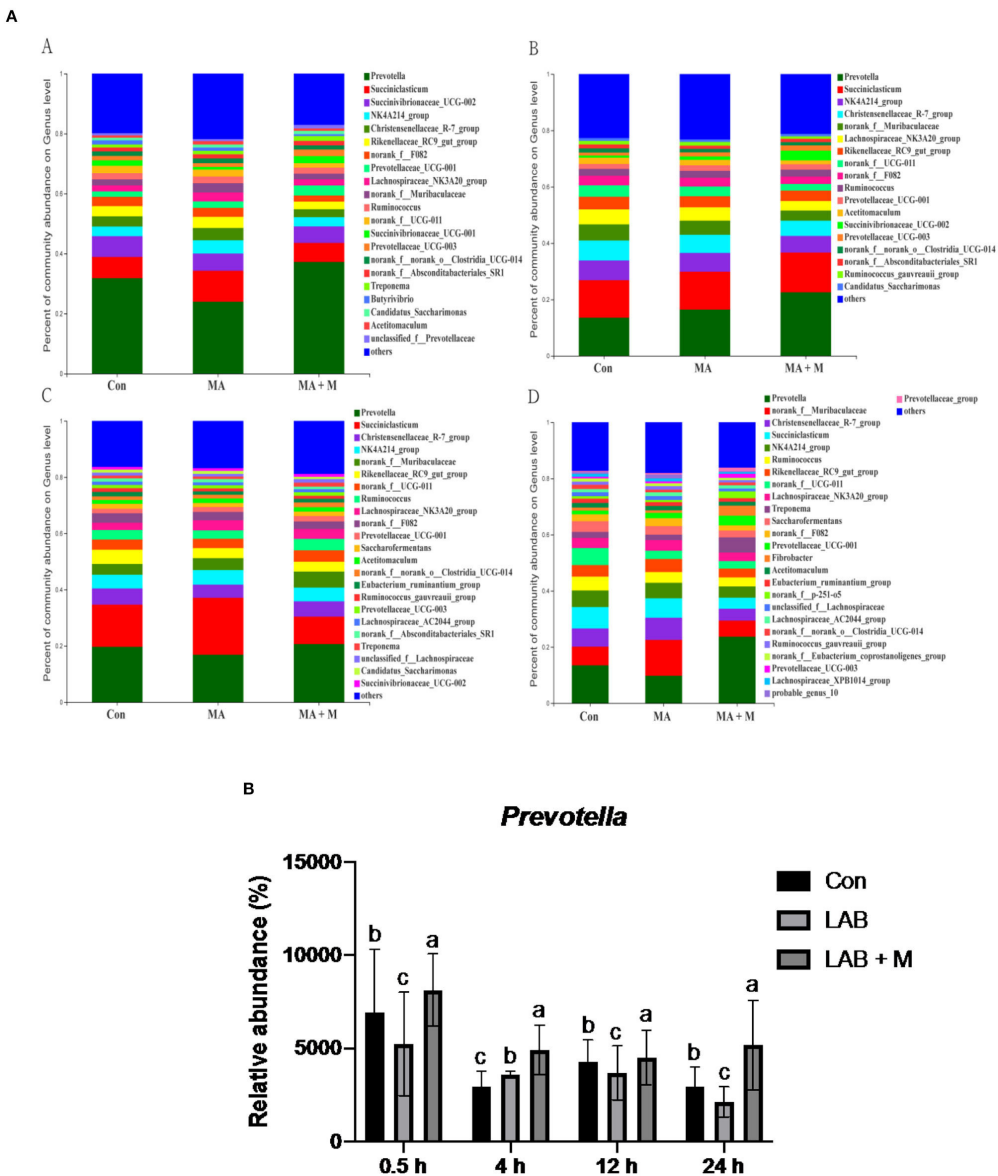
The relative abundance of microbial species under different pretreatment groups. **(A)** Histogram of the relative abundances of species of the rumen microbiome in all pretreatments at the genus level (abundances <0.01 were grouped as “others”). **(B)** The relative abundance of *Prevotella* in all pretreatments. Con, no additive, control; MA, added microecological agents; MA + M, a combination of microecological agents and molasses. Different superscript letters a, b, and c indicate significantly different values (*P* < 0.05) in different groups and the same or no letters indicate insignificant differences (*P* > 0.05).

**Figure 8:**
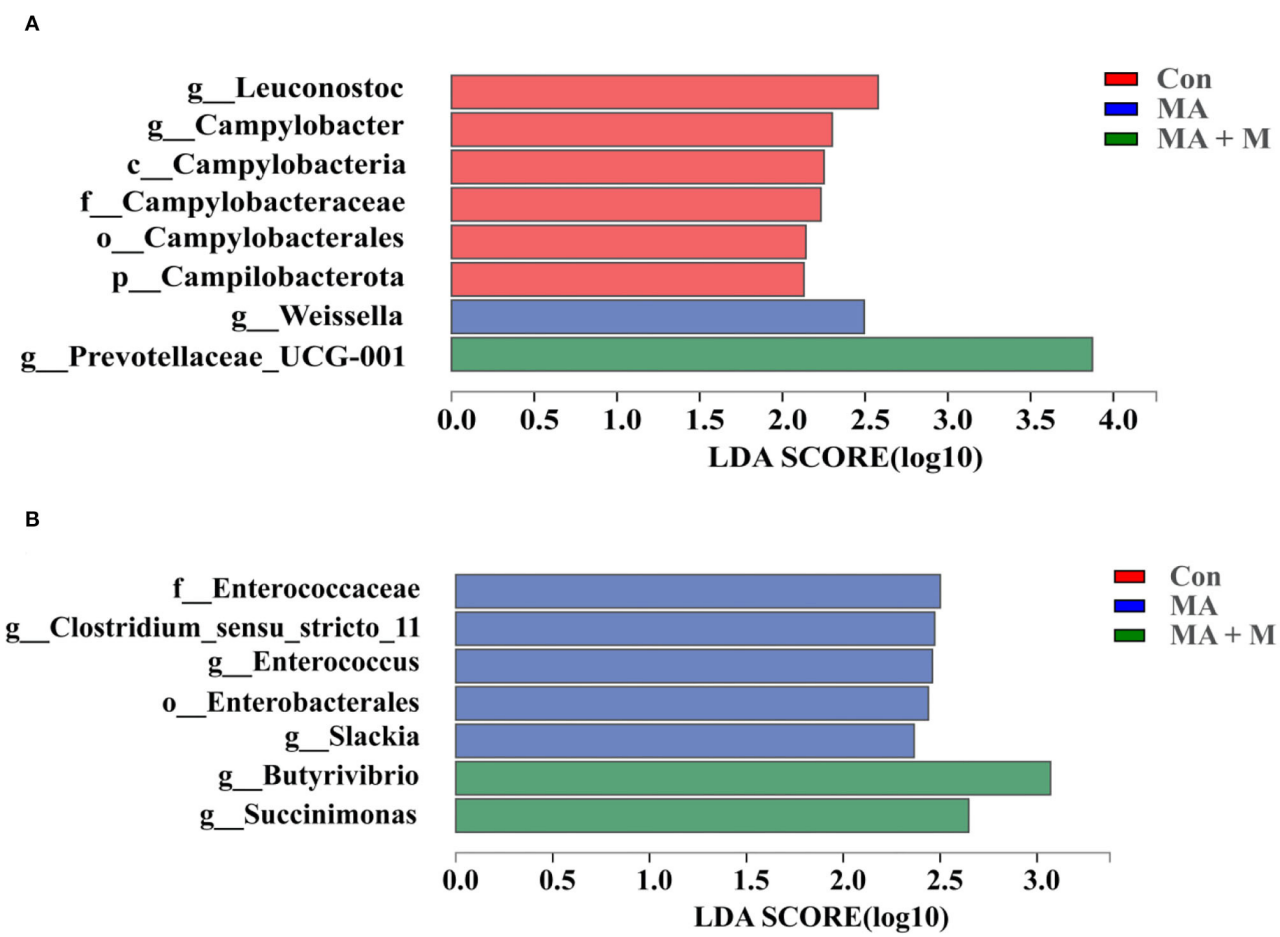
Indicator bacteria with LDA scores of 2 or greater in bacterial communities associated with different pretreatments at 0.5 h **(A)** and 4 h **(B)**. Different-colored regions represent different constituents (red, Con; blue, MA; green, MA + M). Con, no additive, control; MA, added microecological agents; MA + M, a combination of microecological agents and molasses.

We analyzed alpha-diversity by using the Shannon and Chao1 indexes. The results showed no significant differences in all groups (Supplementary Figures S1A,B). Diverse microbial compositions were detected among groups in both phylum and family levels (Figures 6A, 7A). At the phylum level, the *Bacteroidetes* and *Firmicutes* were the most abundant. Rice straw samples were incubated in the rumen for 0.5, 4, and 24 h, and the relative abundance of *Bacteroidetes* was higher (*P* < 0.05) in MA + M than in Con and significantly higher than MA (Figure 6B). Rice straw samples were incubated in the rumen for 4 and 24 h, and the relative abundance of *Firmicutes* was higher (*P* < 0.05) in Con than in other treatments (Figure 6C). At the family level, the *Prevotella* was the most abundant. Rice straw samples were incubated in the rumen for 0.5, 4, 12, and 24 h, and the relative abundance of *Prevotella* was higher (*P* < 0.05) in MA + M than in Con (Figure 7B). Differentially abundant microbial colonization of rice straw sample taxa was further identified by LEfSe analysis. As shown in Figure 8, *Prevotellaceae_UCG-001, Butyrivibrio*, and *Succinimonas* were accumulated in the rice straw with treated MA + M.

### The Link Between Rumen Bacterial Attachment on the Surface of Rice Straw and Environmental Factors

**Figure 9:**
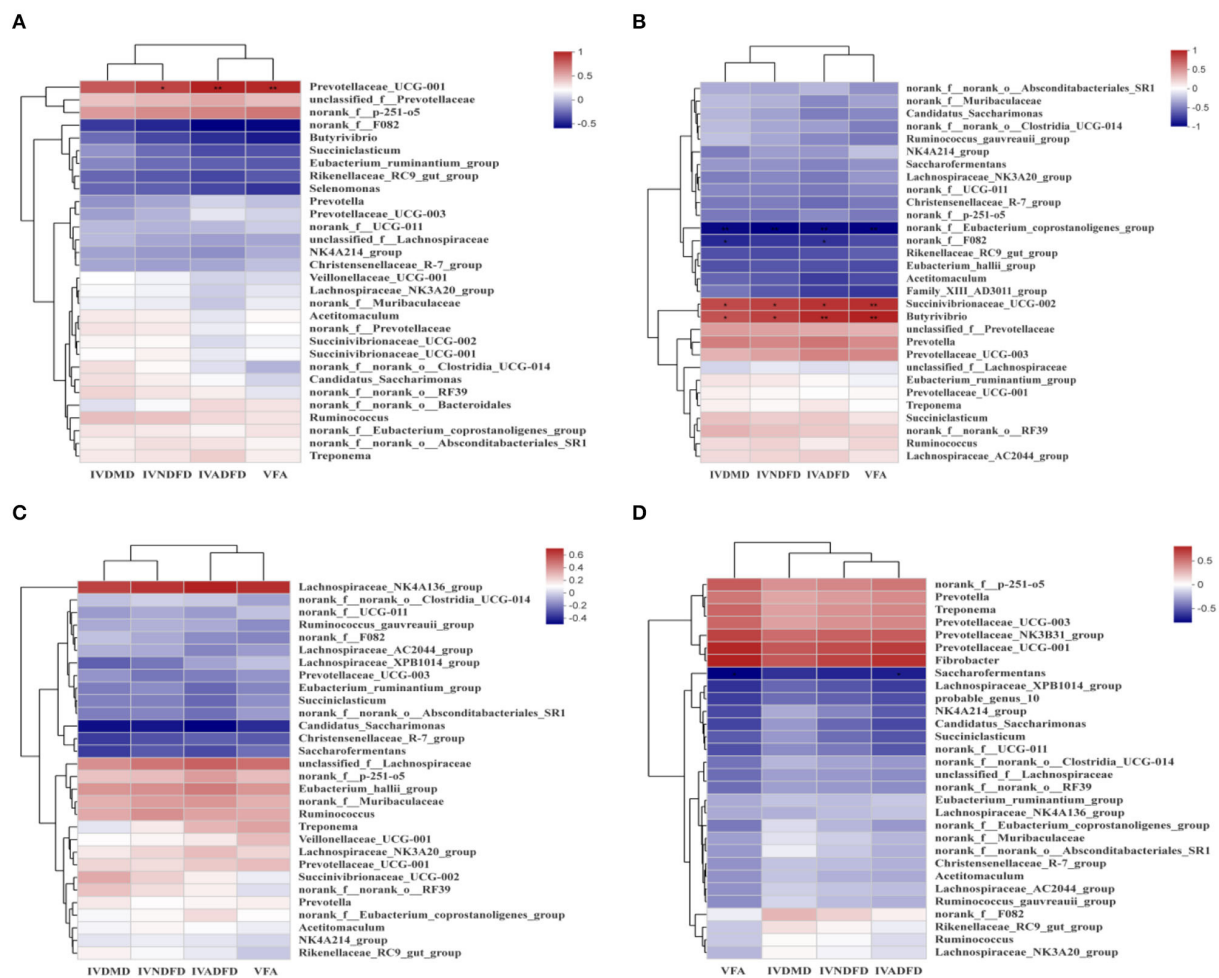
Correlations heatmap of top 30 genera in colonization on surface of rice straw during incubation in rumen 0.5 h **(A)**, 4 h **(B)**, 12 h **(C)**, and 24 h **(D)** and environmental factors. Con, no additive, control; MA, added microecological agents; MA + M, a combination of microecological agents and molasses. *, **, and *** indicate the significant correlations at *P* < 0.05, 0.01, and 0.001. VFA, total volatile fatty acids; IVDMD, *in vitro* dry matter degradability; IVNDFD, *in vitro* neutral detergent fiber degradability; IVADFD, *in vitro* acid detergent fiber degradability.

Analysis the relationship between the colonizated microbes on the surface of rice straw and environmental factors (VFA, IVDMD, IVNDFD, and IVADFD) by Spearman correlation (Figure 9). The relative abundance of *Prevotellaceae_UCG-001* showed a significant positive correlation with VFA (*P* < 0.001) and IVNDFD (*P* < 0.05) and IVADFD (*P* < 0.05) at 0.5 h. And the relative abundance of *Succinivibrionaceae_UCG-002* and *Butyrivibrio* were positively related to the IVDMD (*P* < 0.05), IVNDFD (*P* < 0.05), IVADFD (*P* < 0.05), and VFA (*P* < 0.001) at 4 h, while the relative abundance of *norank_f_Eubacterium_coprostanoligenes_group* was negatively related to the IVDMD (*P* < 0.001), IVNDFD (*P* < 0.001), IVADFD (*P* < 0.001), and VFA (*P* < 0.001) at 4 h.

## Discussion

### Physical Structure and Physicochemical Properties of Pretreated Rice Straw

In this study, we present an optimized novel way to pretreat rice straws. Traditional methods of rice straw management through burning and soil incorporation contribute significantly to environmental pollution. The rice straw novel pretreatment way has overcome not only these limitations but also ensured their efficient utilization as animal feed. Notably, the rumen degradation efficiency of rice straw is an important indicator for pretreatment evaluation. Rumen microbial colonization is very important for feed degradation in the rumen (Pan et al., 2021). However, there are many factors affecting rumen microbial colonization on feed surfaces, including the chemical composition, surface area, and structure of feed (Zhang et al., 2020). Here, our study proved that the untreated rice straw showed a dense and comparatively smooth surface structure. These surface properties can make it difficult for bacteria to bind to the rice straw and colonize it. Nevertheless, after MA or MA + M pretreatment, the rice straw surface became rougher and partially dissolved. This was probably due to the change in hemicellulose from the cellulose and the disruption of fibers by MA or MA + M pretreatment. Previous studies on morphological structure have shown that the corn stover surface becomes rougher and more disordered after the steam explosion treatment (Zhao et al., 2018). Similar structural changes were reported in sugarcane bagasse pretreated with bisulfite (Liu et al., 2017). Furthermore, the internal contents exposed by the surface structure were damaged by MA or MA + M pretreatment, which could promote rumen microbial colonization and improve digestion due to the increased availability of nutrients. The findings indicated that the rice straw treated by MA and MA+ M could provide more colonization sites for rumen microorganisms. Notably, more rumen microbial colonization on the feed surface means higher digestibility (Terry et al., 2020). In addition to the influence of feed surface area on degradation, cellulose CrI is also an important index often used to evaluate the degradation of feed (Chen et al., 2022). Crystallinity is an important parameter to characterize the properties of polymers. The greater the crystallinity, the better the dimensional stability, strength, and heat resistance of the material. Therefore, the study of cellulose crystallinity is very important for the enzymatic digestion of biomass materials (Feuzing et al., 2022). Here, we find that the MA + M decreased the CrI of rice straw. Notably, the negative effect of CrI on the degradation of rice straw has been reported (Gao et al., 2021). This means that MA + M may change the cellulose CrI content of rice straw, which has a positive effect on the release of nutrients. The results were consistent with the recent report that the lower cellulose CrI of Alamo switchgrass contributed to a greater glucose yield (Hu et al., 2011). The results found that the glucan yield of hydrothermally pretreated switchgrass was 38.2% for leaves and 56.3% for internode portions, and their CrI was 48.9 and 46.6%, respectively. Consistently, another study reported that the effective reduction of crystallinity caused by ball milling had a determinant effect on the digestibility of sugarcane bagasse biomass (Da Silva et al., 2010). The results found that glucose yields were 22.0% and 78.7% when the CrI of bagasse was 0.06% and 0, respectively. Jia et al. (2014) documented that corn varieties with low cellulose crystallinity content had higher biomass digestibility. Hence, cellulosic CrI is essential for the enzymatic saccharification of rice straw. In this study, we confirmed that the three spectral profiles of most bands were rather similar. However, the intensities of the absorption peaks showed significant differences, indicating that the basic structure of residual lignin in the MA or MA + M pretreated rice straw samples was not greatly damaged. Some of the chemical bonds in lignin were broken. For example, the intensity of the peak at 3,350 cm^−1^ was reduced after MA or MA + M pretreatment, which corresponded to the O-H stretching of hydrogen bonds of cellulose, hemicellulose, and lignin (Rosa et al., 2012), indicating the partial removal of lignin and hemicellulose from rice straw. The intensity of the peak at 2,900 cm^−1^ was reduced after MA or MA + M pretreatment, which was attributed to C-H stretching within the wax (Iskalieva et al., 2012), showing the removal of wax from rice straw. The band at 1,200–1,000 cm^−1^ was typically related to the C-O-H stretching of cellulose and hemicelluloses. After MA or MA + M pretreatment, the peak at 1,200–1,000 cm^−1^ was reduced, indicating the partial removal of lignin and hemicellulose from rice straw. The band at 1,425 cm^−1^ has been attributed to absorption to C-H deformation within the methoxyl groups of lignin (Guo et al., 2008). After MA or MA + M pretreatment, the peak at 1,425 cm^−1^ was reduced, demonstrating the partial removal of lignin. The intensity of the peak at 1,640 cm^−1^ was reduced after MA or MA + M pretreatment, which was attributed to C=O groups in the alkyl groups of lignin side chains. It was suggested to conjugate with the aromatic structure (Zhao et al., 2010), representing the ether bonds of the lignin structure were hydrolyzed by MA or MA + M pretreatment.

Importantly, the NDF content of feed affects feed intake and ADF affects digestibility (Wang et al., 2021). Here, our study proves that MA or MA + M pretreatments decreased the NDF and ADF of rice straw, which suggests that the rice straw pretreated by MA or MA+ M had a positive effect on digestion. Consistently, Zhang et al. (2020) reported that *in vitro* digestibility improved rice straw by reducing NDF and ADF contents (Yang et al., 2018). Similarly, a study also found that cellulase could reduce the ADF and NDF contents in king grass silages, and the NDF and ADF contents of silages were reduced by 5 and 3% after cellulose treatment (Mao et al., 2014). Zhang Q. et al. (2016) documented that the addition of LAB reduced the ADF and NDF contents in *L. chinensis* silage. The pH value is one of the key indexes to measure the quality of fermentation during silage fermentation (Kholif et al., 2021). The low pH value during silage fermentation means that the activity of harmful microorganisms was inhibited and reduced the loss of nutrients (Fan et al., 2021). In this study, we confirmed that MA + M pretreatment reduced the pH value of rice straw. It was suggested that rice straw treated by MA+ M could improve nutrition quality. Similar studies show that the additive LAB can reduce the pH value of oat silage; after 30 days, the pH value decreased from 4.55 to 4.18 (Cheng et al., 2022). Furthermore, the lower pH value inhibits *C. butyrate* in rice straw silage and inhibits nutrient loss (Tian et al., 2020). During the silage process, organic acids accumulate gradually, which in turn lowers the pH. A study has documented that a pH of 4.2 of silage or below shows well-fermented silage (De Bellis et al., 2022). From the results of this study, it can be observed that the pH value of the MA + M group is lower than the MA group, which may be due to the soluble carbohydrates being lower in rice straw. It needs to be added exogenously in the form of molasses to ensure the rapid reproduction of LAB to produce more organic acids and reduced pH value.

### *In vitro* Ruminal Degradation and Total Gas Production

The nutritional value of feed is mainly determined by the digestibility of ruminants. The *in vitro* culture has become a commonly used technique due to its high correlation with *in vivo* digestibility and ease of operation. Importantly, *in vitro* degradation is one of the most direct indicators to evaluate feed quality, which not only reflects the utilization status of nutrients but also evaluates the effect of pretreatment (Ciriaco et al., 2021). The chemical composition of forage affects digestibility, and generally high CP and low plant cell wall content are beneficial to improving DM digestibility. To understand how MA or MA + M pretreatments affect *in vitro* degradation (IVDMD, IVNDFD, and IVADFD) accurately, we compared the *in vitro* degradation characteristics among Con, MA, and MA + M groups, and as expected, MA or MA + M could improve IVDMD, IVNDFD, and IVADFD of rice straw. The improved degradability of rice straw after MA or MA+M pretreatment is due to the destruction of the cell wall structure of rice straw and the improvement of enzymatic hydrolysis efficiency. These findings suggest that the rice straw pretreated by MA or MA + M could provide more nutrients that can be absorbed and utilized by ruminants (Hymes-Fecht and Casper, 2021) and also imply a positive impact on animal performance (Mpanza et al., 2020). A previous study has also shown that the *L. plantrum* additives can improve *in vitro* degradation of elephant grass (Shah et al., 2021), and the IVDMD and IVNDFD increased by 13.97 and 32.69%, respectively. Similar findings were reported in forage-sorghum silage that the additive LAB can improve the *in vitro* digestibility of oat silage (Kaewpila et al., 2021b). Cherdthong et al. (2020) also reported that *L. casei* TH14 could improve the *in vitro* digestibility of rice straw silage (Cherdthong et al., 2020). Nevertheless, the improved *in vitro* digestibility of MA or MA + M pretreated rice straw is consistent with the lower crystallinity of cellulose and ADF content.

Total gas production is a visual representation of the fermentation degree of feed in the rumen (McIntosh et al., 2003). The greater the degree of fermentation of the feed in the rumen, the greater the gas production. A large amount of gas production indicates that the activity of the rumen microorganisms is high and the fermentation of the substrate is more sufficient; if the gas production is low, it is due to insufficient microbial fermentation products in the substrate (Guo et al., 2022). In fact, gases such as methane, hydrogen, and carbon dioxide are produced by rumen microbes that degrade carbohydrates and other nutrients in the feed (Aragadvay-Yungán et al., 2021). Cumulative gas production can reflect the substrate utilization degree and nutrient value of rumen microorganisms (Sookrali and Hughes, 2021). In this study, we observed that MA + M could improve the GP_72_ of rice straw. Similarly, a study reported that the additive LAB can improve the gas production of corn stover silage (Huang et al., 2022), and the cumulative GP_72h_ increased from 84.49 to 118.19 ml/g. Kaewpila et al. (2021b) also reported that additive LAB to forage-sorghum silage could increase gas production.

The VFA is a critical energy source for the production of Holstein cows. Their composition, yield, and proportion in rumen fluid are important indicators for evaluating rumen fermentation function. The acetate and butyrate of VFA are materials for milk fat synthesis (Chen et al., 2021), and among them, propionic acid glyconeogenesis is the raw material for lactose production improvement (Reynolds, 2006). Higher production of propionate and lower production of acetate and butyrate leads to improved energy efficiency (Knapp et al., 2014). Notably, ruminants ferment the ingested feed in the rumen, and the fermentation products (short-chain fatty acids) are absorbed and utilized by rumen epithelial cells (Johnson et al., 2019). In this study, we confirmed that MA and MA + M pretreatments promoted acetic acid and total VFA production by improving the rice straw nutritional quality. This will help the rumen epithelium to absorb more energy and thus improve the production performance. A recent study has also confirmed that LAB inoculants in corn silage can improve the total VFA concentration in the rumen (Monteiro et al., 2021), compared with the control group, the increase was 13.7%. Kholif et al. (2021) also reported that the addition of LAB to date palm leaves could increase the total VFA in the rumen. Similar studies were reported in wheat straw silage that the additive LAB can improve the acetic acid and total VFA concentration of wheat straw silage (Babaeinasab et al., 2015).

### Microbial Colonization in Pretreated Rice Straw

Ruminants have a special ability to digest and convert plant cell wall polysaccharides into meat and milk. The evolution of the symbiotic interaction between the host and the complex microbial community inhabiting the rumen is responsible for this ability (Grilli et al., 2013). The host depends on a series of enzyme syntheses by these rumen microbes to change the complex fibrous substances into VFA and microbial proteins, which are helpful for growth, production, and maintenance (Kim et al., 2016). The attachment of rumen microorganisms is critical for the establishment of microbial communities associated with feed digestion (Monteiro et al., 2022). Feed entering the rumen is rapidly colonized by microbes followed by the digestion of plant cell wall carbohydrates. Rumen bacteria preferentially colonize damaged areas of plant surfaces (Gharechahi et al., 2020). After the feed is ingested, rumen bacteria play an important role in digestion, fermentation, and degradation. Thus, understanding how MA or MA + M pretreatments affect microbial colonization on the rice straw surface is very important for evaluating the pretreatment effect. We compared the microbial colonization on the rice straw surface among the Con, MA, and MA + M groups by real-time PCR technical. Although there were no differences in the three groups at the incubation for 0.5 and 4 h, there was an obvious increase in microbial colonization on the rice straw surface in the MA + M group during the incubation for 12 and 24 h. The previous study has demonstrated that rumen microbial colonization on the feed surface increases with incubation time and that the chemical composition and structure of the feed strongly influence microbial colonization (Liu et al., 2016). The increased microbial colonization of MA + M pretreated rice straw is consistent with the lower crystallinity of cellulose, NDF, and ADF content. Especially, the destruction of straw surface in the previous result in the MA + M group provided more space for microbial colonization. Notably, improved colonization of microbes on the surface of the MA + M is consistent with increased *in vitro* gas production and *in vitro* digestibility. Similar studies in corn stover reported that the microbial colonization was increased by increasing the porosity and surface area after steam explosion treatment (Sun et al., 2017). These results suggest that the rice straw pretreated by MA + M could improve the degradation performance.

### Diversity of the Bacterial Microbiota Attached to Rice Straw Samples After 0.5 h of Rumen Incubation

Many experiments have demonstrated that rumen microbes rapidly colonized on the surface of ingested feed particles (Piao et al., 2015; Huws et al., 2016), and the composition of colonizing microbial communities is affected by the incubation time, the physical structure, and chemical characteristics (Li et al., 2017). Furthermore, it has been suggested that restricted plasmin access to target substances, and in the rumen the high throughput of lignocellulosic biomass, resist the ability of degradation of plant cell walls in the rumen (Terry et al., 2019). Understanding the limiting steps and mechanisms by which rumen microbes degrade plant cell walls is critical for developing strategies to improve feed utilization in ruminants. To further evaluate the taxonomy and structure of rumen microorganisms colonized on rice straw surfaces accurately, we compared the bacterial compositions among the Con, MA, and MA + M groups. Although there were no differences in the Shannon and Chao indexes in the Con, MA, and MA + M groups, obvious alterations in microbiome structures were detected. Notably, members of the rumen bacterial community have different attachment preferences to rumen particles and the rumen wall (Cheng et al., 2017), and they contribute to nutrient acquisition, maintaining health, and improving production (Liu et al., 2016). Here, the findings of this study indicated that MA or MA + M promoted the degradation of rice straw by altering the compositions of rumen microbial colonization on the surface of rice straw, including a severe reduction in the abundance of *Firmicutes*, and an increase in the abundance of *Bacteroidetes, Prevotella, Prevotellaceae_UCG-001, Butyrivibrio*, and *Succinimonas*. During the whole process of rumen incubation, *Bacteroidetes* were significantly enriched in the MA + M group. Particularly, the colonization of rumen microbes is caused by microbial populations with distinct roles that alter with time (Cao et al., 2022). It has been documented that the *Bacteroidetes* show the dominant epiphytic community colonizing ryegrass and ryegrass hay (Belanche et al., 2017). In a previous study, it has been shown that the members of *Bacteroidetes* respond primarily to proteolysis and carbohydrate degradation (Chen et al., 2015). The *Bacteroidetes* are among the most abundant members of the rumen microbiota and function to degrade carbohydrates, and their genomes show good lignocellulose degradation ability due to the presence of a large number of pectinolytic enzymes and cellulolytic enzymes (Lapébie et al., 2019). Similarly, *Prevotella* spp. is an abundant member of the rumen microbiota with the ability to grow on substrates such as cellulose, hemicellulose, and pectin (Golder et al., 2014). Their main properties are to degrade feed xylan in the rumen and thus play an essential role in fiber degradation (Dodd et al., 2010). In this study, we observed that the *Prevotella* significantly enriched in MA + M pretreated rice straw, implicating MA + M pretreated rice straw had a greater degradation in the rumen. Notably, according to our previous results, rice straw treated with MA + M not only increased the concentration of VFA but also suffered a large degree of structural damage, which means that rumen microbial accessibility of carbohydrates may be increased. The enrichment of *Prevotella* is consistent with the increased accessibility of simple carbohydrates (Beauchemin et al., 2019). *Prevotella* is involved in the metabolism of carbohydrates and nitrogen and can break down sugars and break down cellulose (Kim et al., 2017). Furthermore, the application of LEfSe revealed differential pathways between Con, MA, and MA + M. We observed that *Butyrivibrio, Prevotellaceae_UCG-001*, and *Succinimonas* were enriched in MA + M pretreated rice straw. This is consistent with previous studies that report *Butyrivibrio* and *Succinimonas* as having an important role in forage degradation (Chesson et al., 1986; Krause et al., 2003). Such findings are similar to the changes in alfalfa hay degradation in the rumen (Liu et al., 2016). Our results demonstrated that the MA + M could induce rumen microorganisms with lignocellulosic degradation ability to colonize rice straw surfaces.

Furthermore, the relationship between microbial colonization on the surface of rice straw treated by MA or MA + M and *in vitro* degradation was analyzed. We observed that *Prevotellaceae_UCG-001, Succinivibrionaceae_UCG-002*, and *Butyrivibrio* were positively related to IVDMD, IVNDFD, IVADFD, and VFA. These results clearly prove that rice straw treated by MA or MA+ M could induce the colonization of rumen microorganisms with fiber degradation ability, thus improving the degradation of rice straw in the rumen. In addition, we have summarized the effects of different pretreatments on different parameters of rice straw (Table 4).

**Table 4:** Comparison among different studies using various pretreatment methods for rice straw.

**Pretreatments**	**Species**	**Chemical changes**	**CrI (%)**	**Degradation**	**Rumen microorganism**	**References**
Compound enzyme preparation	Rice straw	Lignin content reduced by 1.29%	CrI reduced by 7.34%	–	–	Yu-Rong et al., 2017
Compound enzyme preparation + *Lactobacillus plantarum* + *Lactobacillus buchneri*	Corn stover	NDF and ADF decreased by 19.9 and 11.2%, respectively	CrI increased by 2.85%	–	–	Jianhong et al., 2018
*Bacillus amyloliquefaciens* HRH317 and *Bacillus subtilis* CP7	Corn silage	NDF and ADF decreased by 1.3 and 1.7%, respectively	–	–	–	Bai et al., 2022
Probiotic	Sorghum vegetable silage	–	–	DMD increased by 7.3%	Increased abundance of *Prevotella*	Forwood et al., 2020
*Lactobacillus plantarum*	Alfalfa silage	NDF decreased by 1.1%	–	–	–	Li et al., 2020
*Lentilactobacillus buchneri*	Oat Silage	DM loss reduced by 1.28%	–	–	–	Cheng et al., 2022
Lactic acid bacteria	Alfalfa silage	DM loss reduced by 3.04%	–	–	–	Ergin and Gumus, 2020
Lactic acid bacteria	Whole crop corn silage	NDF and ADF decreased by 12.3 and 9.4%, respectively	–	DMD increased by 6.1%	–	Nair et al., 2020
Lactic acid bacteria	Forage sorghum silage	NDF and ADF decreased by 10.03 and 8.15%, respectively	–	IVDMD increased by 6.85%		Kaewpila et al., 2021a
Compound enzyme preparation + *Lactobacillus plantarum* + *Lactobacillus brucella*	Rice straw	–	–	DMD increased by 15.56%	–	Xiaowen et al., 2016
Compound enzyme preparation + *Lactobacillus plantarum* + *Lactobacillus brucella*	Corn stover	NDF decreased by 4.9%	–	NDFD increased by 7.99%	–	Lian et al., 2016

*Compound enzyme preparation: Cellulase + xylanase + β -glucase, pectinase, laccase. CrI (%), Crystallinity of cellulose; DM, dry matter; NDF, neutral detergent fiber; ADF, acid detergent fiber; DMD, dry matter degradation; IVDMD, in vitro dry matter degradation; NDFD, neutral detergent fiber degradation*.

## Conclusion

Altogether, we concluded that the MA or MA + M pretreated rice straw significantly reduces NDF and ADF contents but also increases CP content. Based on chemical composition and fermentation quality, 30 days was the optimal duration for pretreatment of rice straw as ruminant feed. The MA or MA + M destruct the rice straw's structure and increases its surface area, which leads to the enhancement of microbial colonization, fermentation, and fiber digestion. Furthermore, MA + M specifically induced the colonization of *Prevotellaceae_UCG-001, Butyrivibrio*, and *Succinimonas* of rumen on the surface of rice straw. In this study, we used a novel MA to pretreat the refractory rice straw and analyzed the reasons for the improved degradation after pretreatment in terms of changes in physicochemical structure and specific induction of rumen microbial colonization. Microecological agents and molasses combined pretreatment provided a new strategy for the pretreatment of lignocellulosic raw materials in the future and thus could be useful in the alleviation of the shortage of ruminant feed resources.

## Data Availability Statement

16S rRNA gene data has been deposited in the National Center for Biotechnology Information (NCBI) Sequence Read Archive (SRA) database under accession number PRJNA796439 and PRJNA838404. The links are https://dataview.ncbi.nlm.nih.gov/object/PRJNA796439 and https://dataview.ncbi.nlm.nih.gov/object/PRJNA838404.

## Ethics Statement

This study was reviewed and approved by the Animal Care Committee of the College of Animal Science and Technology of China Agriculture University (Protocol number: 2013-5-LZ).

## Author Contributions

ZC, YM, and XC mainly designed this experiment. YM and XC conducted an animal experiment. Data were collected and analyzed by XC and YM. The manuscript was mainly written by YM and edited by MK, JX, and ZC. All authors contributed to the article and approved the submitted version.

## Funding

This study was supported by the 2115 Talent Development Program of China Agricultural University Beijing, P. R. China) and the Zhulin Agriculture Co., Ltd. (Gushi, Henan, P. R. China).

## Conflict of Interest

The authors declare that the research was conducted in the absence of any commercial or financial relationships that could be construed as a potential conflict of interest.

## Publisher's Note

All claims expressed in this article are solely those of the authors and do not necessarily represent those of their affiliated organizations, or those of the publisher, the editors and the reviewers. Any product that may be evaluated in this article, or claim that may be made by its manufacturer, is not guaranteed or endorsed by the publisher.
