# Supplementary material for: A Combination of Novel Microecological Agents and Molasses Role in Digestibility and Fermentation of Rice Straw by Facilitating the Ruminal Microbial Colonization
Source: Front Microbiol. 2022 Jul 14;13:948049. doi: 10.3389/fmicb.2022.948049 (PMC9329086; doi:10.3389/fmicb.2022.948049)
Supplement: Supplementary file 1 [file Data_Sheet_1.PDF]

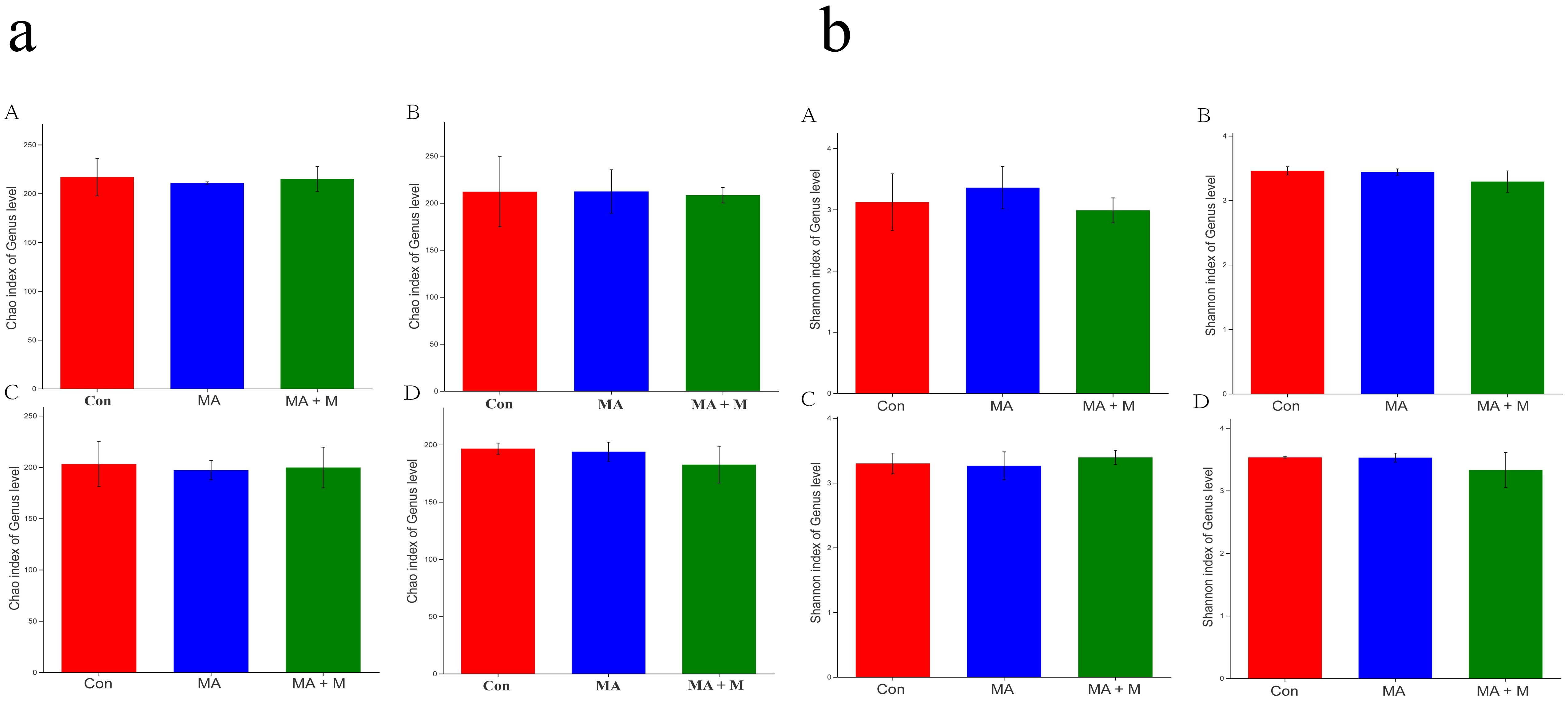

Figure S1: α-diversity of different groups after incubation in rumen 0.5 h (A), 4 h (B), 12 h (C) and 24 h (D) by chao1 and shannon index in genus level. Con: no additive, control; MA: added microecological agents; MA + M: a combination of microecological agents and molasses; Data were mean ± SEM. *P* values were determined using the t-test. **P*≤0.05, ***P*≤0.01.
